# West Nile virus: a newly emergent epidemic disease.

**DOI:** 10.3201/eid0707.017715

**Published:** 2001

**Authors:** V Deubel, D J Gubler, M Layton, M Malkinson

**Affiliations:** Institute Pasteur, Paris, France.


### West Nile Virus: A Newly Emergent Epidemic Disease

**Vincent Deubel,* Duane J. Gubler,† Marcelle Layton,* and Mertyn Malkinson‡**
*Institute Pasteur, Paris, France; †Centers for Disease Control and Prevention, Fort Collins, Colorado, USA; and ‡Kimron Veterinary Institute, Beit Dagan, Israel

West Nile (WN) virus is a mosquitoborne virus of the genus *Flavivirus*, family *Flaviviridae*. WN, Japanese encephalitis, St. Louis encephalitis, Murray Valley encephalitis, and Kunjin viruses (along with other viruses) belong to the Japanese encephalitis serocomplex and are closely related to each other genetically and ecologically. The Japanese serocomplex of viruses has a near global distribution with some overlap. All the viruses are maintained in cycles involving birds as vertebrate hosts and mosquitoes (principally *Culex* species) as vectors.

WN virus has a wide geographic distribution in Africa, west and central Asia, the Middle East, and Europe. Historically, epidemics have been infrequent and not associated with severe disease. In the past decade, however, epidemics/epizootics have occurred in several countries, including Romania (1996, humans), Morocco (1996, horses), Tunisia (1997, humans), Italy (1998, horses), Israel (1997, 1998, 1999, domestic geese), Russia (1999, birds and humans), and the United States (1999, humans, birds, and horses). Severe neurologic disease and fatalities have occurred in all these outbreaks.

The 1999 epidemic/epizootic in New York affected humans (62 laboratory-positive cases of neurologic disease with 7 deaths), birds (thousands of bird deaths, with illness and death documented in 26 species), and horses (25 cases with 9 deaths). The epicenter of the outbreak was the Queens section of New York City, where more than half of the laboratory-positive human cases occurred. The outbreak in humans peaked in late August, with the first patient experiencing onset of symptoms on August 2, and the last on September 22. Virus transmission became widespread, and WN virus–positive birds, mosquitoes, or both ultimately were documented in New York, Connecticut, New Jersey, and Maryland. Virus isolation data suggest the 1999 outbreak was transmitted by *Culex* species mosquitoes, principally *Culex pipiens*. Overwintering mosquitoes of this species collected in January and February 2000 were found to be positive for WN virus.

Comparison of sequenced virus isolates from birds, horses, and mosquitoes and viral sequences amplified from human brain tissue in the 1999 New York outbreak confirmed that the viruses infecting all species were identical. Moreover, these viruses were identical (99.9% nucleotide homology) to a virus isolated in 1998 from domestic geese in Israel. Analysis of amino acid and nucleic acid sequences for a portion of the envelope gene of 40 WN virus strains from wide geographic areas produced pylogenetic trees showing that WN viruses segregate into two lineages. Lineage I includes viruses from Africa, all strains from north Africa, Europe, Israel, the United States, and Kunjin virus from Australia. Lineage II is composed only of strains from west, central, and east Africa and Madagascar. The data strongly suggest that the virus causing the 1999 New York epidemic/epizootic was introduced from Israel or the Middle East.

The epizootics in domestic geese in Israel over 3 years (1997, 1998, and 1999) and the strong genetic similarity among WN virus strains suggest that the virus may have persisted in the area in mosquitoes, ticks, or chronically infected birds. Alternatively, WN virus could have been reintroduced in migrating birds from Africa or from Europe. Israeli data provide strong evidence that WN virus is introduced in white storks. It is possible that both mechanisms are correct.

The recent epidemics/epizootics of WN virus in north Africa and Europe and the unprecedented epidemic/epizootic in the northeastern United States underscore the ease with which exotic pathogens can move between continents and regions today. These epidemics/epizootics also reinforce the need to rebuild the public health infrastructure to deal with epidemics of vectorborne diseases and to develop effective surveillance, prevention, and control strategies for these diseases.

Address for correspondence: Duane J. Gubler, Centers for Disease Control and Prevention, Foothills Campus, P.O. Box 2087, Fort Collins, CO 80522 USA; fax: 303-221-6476; e-mail: djg2@cdc.gov

